# Detection of the arcuate fasciculus in congenital amusia depends on the tractography algorithm

**DOI:** 10.3389/fpsyg.2015.00009

**Published:** 2015-01-21

**Authors:** Joyce L. Chen, Sukhbinder Kumar, Victoria J. Williamson, Jan Scholz, Timothy D. Griffiths, Lauren Stewart

**Affiliations:** ^1^Department of Physical Therapy and Graduate Department of Rehabilitation Sciences, Heart and Stroke Foundation Canadian Partnership for Stroke Recovery, Sunnybrook Research Institute, University of TorontoToronto, ON, Canada; ^2^Wellcome Trust Centre for Neuroimaging, Institute of Neurology, University College LondonLondon, UK; ^3^Institute of Neuroscience, University of Newcastle Upon TyneNewcastle Upon Tyne, UK; ^4^Department of Music, University of SheffieldSheffield, UK; ^5^Mouse Imaging Centre, The Hospital for Sick ChildrenToronto, ON, Canada; ^6^Department of Psychology, Goldsmiths, University of LondonLondon, UK

**Keywords:** arcuate fasciculus, congenital amusia, diffusion magnetic resonance imaging, tractography, deterministic, probabilistic, crossing fibers

## Abstract

The advent of diffusion magnetic resonance imaging (MRI) allows researchers to virtually dissect white matter fiber pathways in the brain *in vivo*. This, for example, allows us to characterize and quantify how fiber tracts differ across populations in health and disease, and change as a function of training. Based on diffusion MRI, prior literature reports the absence of the arcuate fasciculus (AF) in some control individuals and as well in those with congenital amusia. The complete absence of such a major anatomical tract is surprising given the subtle impairments that characterize amusia. Thus, we hypothesize that failure to detect the AF in this population may relate to the tracking algorithm used, and is not necessarily reflective of their phenotype. Diffusion data in control and amusic individuals were analyzed using three different tracking algorithms: deterministic and probabilistic, the latter either modeling two or one fiber populations. Across the three algorithms, we replicate prior findings of a left greater than right AF volume, but do not find group differences or an interaction. We detect the AF in all individuals using the probabilistic 2-fiber model, however, tracking failed in some control and amusic individuals when deterministic tractography was applied. These findings show that the ability to detect the AF in our sample is dependent on the type of tractography algorithm. This raises the question of whether failure to detect the AF in prior studies may be unrelated to the underlying anatomy or phenotype.

## Introduction

Diffusion magnetic resonance imaging (MRI) is widely used to virtually dissect white matter fiber pathways in the brain. In the case of failed tracking the question arises as to whether this might have a biological explanation or reflect limitations of the tracking algorithm used (Dell'Acqua and Catani, 2012; Campbell and Pike, 2014). In this study we address the basis for the failure to track a specific white matter tract in a biological disorder: the right arcuate fasciculus (AF) in congenital amusia or tone deafness.

Individuals with amusia lack the ability to process aspects of music in the absence of other neurological impairment, learning difficulty or hearing loss (Ayotte et al., 2002). These individuals have poor pitch perception (Foxton et al., 2004; Hyde and Peretz, 2004) and pitch memory (Gosselin et al., 2009; Tillmann et al., 2009; Williamson and Stewart, 2010). Some individuals also have relatively preserved pitch production abilities as compared to pitch perception (Loui et al., 2008, 2009; Dalla Bella et al., 2009; Hutchins and Peretz, 2013). However, the extent and direction of such a perception-production dissociation may be related to individual behavioral differences and task demands (Williamson et al., 2012).

Studies of cortical anatomy in amusia show abnormalities in the right inferior frontal cortex and superior temporal gyrus (STG) (Hyde et al., 2006, 2007; Albouy et al., 2013), with reduced neural activity in the former and reduced functional connectivity between these regions (Hyde et al., 2011). Abnormal effective connectivity between these regions has also been demonstrated using dynamic causal modeling (Albouy et al., 2013). From first principles, deficits in effective connectivity might be based on a physical disconnection caused by structural change in a fiber tract, or be due to another cause in the presence of preserved anatomy. The former has been hypothesized in work suggesting the reduced volume or absence of the right AF may underlie perception-production impairments exhibited by amusics (Loui et al., 2009).

The AF is a tract that connects auditory with motor brain regions (Petrides, 2013) and shows asymmetries that are plausibly related to language lateralization (Friederici, 2009). A number of studies have reported the inability to track the AF in the left (Glasser and Rilling, 2008; Lebel and Beaulieu, 2009) and right (Catani et al., 2007; Glasser and Rilling, 2008; Lebel and Beaulieu, 2009; Kaplan et al., 2010; Thiebaut de Schotten et al., 2011) hemispheres in some healthy individuals. These studies all used deterministic tracking without the modeling of multiple fibers, and in fact, two of these studies suggest that failure to detect the AF may be due to limitations of this algorithm (Glasser and Rilling, 2008; Kaplan et al., 2010). For example, Glasser and Rilling (2008) state that the AF was identified in all subjects in another study they conducted using probabilistic tractography with crossing fiber modeling (Rilling et al., 2008).

One study that tested individuals with amusia showed the right AF, connecting posterior STG with pars opercularis, could not be tracked in nine out of 10 participants (Loui et al., 2009). This study also used deterministic tractography without modeling crossing fibers. In light of findings where the AF is undetected in some neurologically normal controls, it remains to be verified whether the disconnection syndrome in amusia is in fact related to their behavioral phenotype. Therefore, the present study investigates the extent to which the ability to detect the left and right AF in amusic and control individuals depends on the type of tracking algorithm.

Briefly, diffusion MRI acquires a series of images to measure hindrance to intra- and extra-cellular water diffusion in the brain. Spontaneous water diffusion can be hindered by cellular membranes such as the myelin sheath that wrap the axonal tracts of the white matter (Beaulieu, 2002). Water therefore tends to flow along axons, and its pathway is hindered perpendicular to axons. The local axon orientation estimated from the diffusion data can then be used to track potential white matter pathways in the brain (by connecting neighboring voxels depending on their orientation). In deterministic tractography, “the estimated fiber orientation is assumed to represent the best estimate to propagate streamlines” (Dell'Acqua and Catani, 2012). The tracking algorithm thus follows the same direction each time it passes through the same location. In contrast, probabilistic tractography estimates the uncertainty of likely fiber orientations. Therefore, the orientation varies with each pass depending on its probability distribution (Behrens and Jbabdi, 2009). Both algorithms have their advantages and disadvantages (Dell'Acqua and Catani, 2012; Campbell and Pike, 2014). For example, deterministic algorithms use a fractional anisotropy threshold to prevent tracking into areas of low anisotropy, where noise may dominate the signal. In contrast, probabilistic algorithms can track through these regions, which may lead to false positives. However, probabilistic tractography allows the reconstruction of fibers with lower probability that may be missed by deterministic tractography, and can track through regions of low anisotropy, which again, can improve the reconstruction of fibers (Behrens and Jbabdi, 2009). Most importantly, reconstruction of a fiber pathway using a single fiber model may miss branches of the pathway that cross with other tracts, especially if a competing pathway is stronger (Behrens et al., 2007; Behrens and Jbabdi, 2009). Furthermore, it is estimated that 90% of voxels contain two or more fiber populations, therefore, making the modeling of crossing fibers highly relevant (Jones et al., 2013). This is especially the case when tracking the AF since some of its fibers run in a rostral-caudal direction, crossing or intersecting with the corticospinal tract that runs in a dorso-ventral direction (Behrens et al., 2007).

Given the aforementioned differences between tracking algorithms, we used three tracking algorithms all implemented in the FSL software package (http://www.fmrib.ox.ac.uk/fsl). This allows us to control for differences that may exist between different software packages. The first two models used probabilistic tractography. In the first, 2-fiber model, we modeled crossing fibers. In the second, 1-fiber model, we modeled only one fiber population. The third model used a deterministic tracking approach in which only one fiber orientation per voxel is derived from the first eigenvector or dominant orientation of the diffusion tensor. This model more closely resembles those used in the aforementioned studies (Catani et al., 2007; Glasser and Rilling, 2008; Lebel and Beaulieu, 2009; Loui et al., 2009; Kaplan et al., 2010; Thiebaut de Schotten et al., 2011) where tracking of the AF failed in some cases. Given that the modeling of crossing fibers (e.g., AF and corticospinal tract) is highly relevant and is shown to influence tractography results (Behrens et al., 2007), we hypothesize that the probabilistic 2-fiber model would be more robust to detect the AF compared to the probabilistic 1-fiber and deterministic 1-fiber models.

There exists considerable uncertainty over where the human AF starts and ends, and consequently studies have varied in how they define this tract (Dick and Tremblay, 2012). Non-human primate tracer studies demonstrate that the AF connects caudal STG (area Tpt) and sulcus (area TPO) with dorsal areas 8 and 6 (Petrides and Pandya, 1988, 2002; Schmahmann and Pandya, 2006). Recently, modest connections to areas 44 and 45 have been found (Petrides and Pandya, 2009). Work in human resting state fMRI, also show connectivity patterns that corroborate these tracer studies (Kelly et al., 2010; Margulies and Petrides, 2013). In contrast, human diffusion MRI studies have defined the AF by tracking between various posterior temporal and parietal regions (e.g., STG, middle temporal gyrus (MTG), the inferior parietal lobule) to various frontal regions (e.g., ventral premotor cortex, areas 44 and 45, and middle frontal gyrus) (Catani et al., 2005, 2007; Powell et al., 2006; Barrick et al., 2007; Glasser and Rilling, 2008; Matsumoto et al., 2008; Lebel and Beaulieu, 2009; Loui et al., 2009; Thiebaut de Schotten et al., 2011). Given these apparent differences in monkey tracer studies and human diffusion MRI data, a debate is whether the AF in humans is indeed comprised of these various origin and termination regions (Dick and Tremblay, 2012). A problem with tractography analysis is that it is always possible to delineate a path joining A and B; results are not necessarily constrained by true anatomy. Although one may impose rules to restrict tractography, these are defined by the investigator. Thus, it is suggested that anatomical connectivity studies based on tracers in macaques are the gold-standard (Petrides, 2013). Recent work that combines tracer measurements in macaques with diffusion MRI of both macaques and humans suggests that there is a good correspondence between the techniques (Jbabdi et al., 2013). It was found that the organizational principles of white matter pathways in the ventral prefrontal cortex in macaques are preserved in humans (Jbabdi et al., 2013). However, this has yet been verified for the AF.

Given the controversies (Dick and Tremblay, 2012; Petrides, 2013), we avoid defining the origin and termination points of the AF and virtually dissect the bulk of the tract, following previously established methods (Giorgio et al., 2010). This said, one caveat is it is difficult if not impossible with current diffusion MRI approaches to dissociate the AF from other branches of the superior longitudinal fasciculus (e.g., II and III) that originate from the inferior parietal cortex and terminate in ventrolateral and rostroventral frontal cortex, and the middle longitudinal fasciculus that originate in lateral temporal regions and terminate in the inferior parietal cortex; these fiber pathways all course through the same region (Frey et al., 2008; Petrides, 2013). This is in contrast to macaque tracer studies that are able to successfully dissociate these fiber systems (Petrides, 2013). Thus, our approach also does not dissociate the series of two U-shaped fibers [likely the superior and middle longitudinal fasciculi (Petrides, 2013)] that has been suggested to comprise the right AF (Catani et al., 2007). Therefore, for the purpose of this study, we refer to the AF but acknowledge that the fiber reconstructions may derive from any of the aforementioned fiber pathways. It is not in the scope of the study to compare the specific implementations of the many available tracking algorithms and diffusion models that may influence tractography, nor to determine which of these can best segregate the different fiber pathways. The aim of the study is to compare three commonly used tracking approaches in their ability to detect the bulk of the AF. This will allow us to determine whether prior findings in healthy and amusic individuals that failed to detect the AF, may relate to the tractography approach. As such, this study is not an attempt to precisely replicate prior work given the theoretical (see Discussion above about AF anatomy) and methodological differences (see Materials and Methods).

## Materials and methods

### Participants

Participants were initially tested for congenital amusia using an online assessment (www.delosis.com/listening/home.html) that incorporates the Scale subtest of the Montreal Battery of Evaluation of Amusia (MBEA) (Peretz et al., 2003). Individuals who scored two standard deviations below the mean composite score of a normative sample (Peretz et al., 2003) on two consecutive occasions were invited for a laboratory-based assessment. During this assessment, the three pitch-based tests of the MBEA battery (Scale, Contour and Interval) were administered in a sound attenuated room. For each participant, these scores were summed to generate a pitch-composite score. A criterion cut-off of 65 (2 SD below the mean) was applied to confirm amusia, based on the pitch composite score of a normative sample (Peretz et al., 2003; Liu et al., 2010; Williamson and Stewart, 2010).

We tested 26 participants with amusia and 26 healthy controls. Participants gave written informed consent to participate in the experiments, which were approved by the Ethics Committee at Goldsmiths, University of London. An individual with amusia was tested but excluded from analyses because neuroimaging data subsequently showed this person had abnormally large ventricles. Amusic (*n* = 25) and control individuals were matched on gender, age, number of years of musical education, and Digit Span (Wechsler Adult Intelligence Scale; WAIS) (see Table 1). An independent samples *t*-test showed a significant difference on National Adult Reading Test (NART) score, with amusics scoring lower than controls (*t* = 2.13, *p* = 0.04). The amusic group also had more years of formal education than the control participants (*t* = 2.15, *p* = 0.04). Prior literature has not shown years of formal education and the NART score to explain the amusic phenotype. However, analysis was conducted on the full set of participants as well as subgroups (*n* = 14 amusics, *n* = 15 controls) that were matched on these variables, NART (*p* = 0.07) and years of formal education (*p* = 0.15) (see Supplemental Methods, Table 1). Selection of these subgroups were selected blind to the results of the tractography analyses.

**Table 1:** **Demographics**.

	**N**	**Age**	**NART**	**Digit span**	**Years musical training**	**Years formal education**	**MBEA scale**	**MBEA contour**	**MBEA interval**	**Pitch composite**
Amusic	25									
Mean		51.48	41.35 (*n* = 20)	19.90 (*n* = 20)	4.68	16.21 (*n* = 19)	19.28	20.00	18.20	57.48
SD		10.86	5.71	3.71	0.48	1.51	2.72	2.83	2.12	6.03
Control	26									
Mean		50.46	44.42	20.08	4.58	14.88	27.15	27.73	26.81	81.69
SD		12.02	4.01	3.74	0.50	2.18	2.05	1.85	2.47	5.37
T statistic		0.32	2.13	0.16	0.75	2.15	11.71	11.61	13.34	15.16
*P*-value		0.75	0.04	0.87	0.47	0.04	<0.001	<0.001	<0.001	<0.001

N, number of participants; NART, National Adult Reading Test; MBEA, Montreal Battery of Evaluation of Amusia.

### MRI acquisition

Data were acquired using a 3-Tesla Trio Siemens scanner with a 12-channel head coil. A high resolution T1-weighted structural image was obtained for each participant at 1 × 1 × 1 mm^3^ voxel resolution, *FOV* = 256 × 240 mm^2^, matrix size = 256 × 240 mm^2^, *TE* = 2.48 ms, *TR* = 7.92 ms, flip angle = 16°. Diffusion MRI was also performed and images for each participant were acquired at 2.29 × 2.29 × 2.30 mm^3^ resolution (matrix size = 96 × 96 mm^2^, *FOV* = 220 × 220 mm^2^, slice thickness = 2.3 mm, 60 slices, *TR* = 15000 ms, *TE* = 90 ms, flip angle = 90°, Partial Fourier Imaging = 6/8). One set of 60 diffusion weighted images was acquired with a *b*-value of 1000 s/mm^2^, as well as 6 no diffusion weighted images. Data were collected with reversed phase-encode blips, resulting in pairs of images with distortions going in opposite directions. From these pairs the susceptibility-induced off-resonance field was estimated (Andersson et al., 2003) as implemented in FSL (Smith et al., 2004) and the two images were combined into a single corrected one.

### MRI data analysis

All analyses were performed using FSL tools (release 4.1). The diffusion data were preprocessed using FMRIB's Diffusion Toolbox (FDT) and *topup* (Andersson et al., 2003). The data were corrected for head motion, eddy current and susceptibility-induced distortions, and brain extracted using *BET*.

*Bedpostx* fits the probabilistic diffusion model on the corrected data. *Bedpostx* was performed by fitting 2 fibers per voxel, which allows for the modeling of crossing fibers. In addition, *bedpostx* was also implemented using a 1-fiber model. For the deterministic tracking the diffusion tensor was estimated using *dtifit*. The orientation of the first eigenvector was converted to the spherical coordinate system and used as the only sample of the orientation distribution.

Tractography of the AF was then performed using *probtrackx* with 5000 samples. Three different tractography analyses were performed. The first used inputs from the default 2-fiber model from *bedpostx* and the second used inputs from the 1-fiber model. In both of these analyses, probabilistic tractography was performed using the default settings in FDT. The third tractography analysis simulated deterministic tracking by working on a one-sample orientation distribution. By using *probtrackx* for all three scenarios we ensured that the results only depended on the degree of fiber orientation modeling and not on subtle variations in interpolations, step sizes, and stopping criteria.

For all three analyses, tractography of the AF was constrained using a multiple region of interest (ROI) approach. These ROIs were identical to the ones implemented by Giorgio et al. (2010) drawn in standard MNI152 space and implemented in the same fashion across all three analyses. The crucial difference in how the AF is defined in this paper as compared to that of Loui et al. (2009) is that we dissect the central white matter portion of the tract. We do not track to white matter that underlies gray matter origin and termination points of the AF. This is because the precise location of these regions is of considerable debate in the literature, as discussed in the Introduction. Our approach is able to capture all fibers considered to be part of the AF that are assumed to originate and terminate in any of the hypothesized areas. Furthermore, the ROIs in the study of Loui et al. (2009) were drawn in each individual's fractional anisotropy image, thus no standard (or individual) coordinates were provided to enable an exact replication of their method.

**Figure 1:**
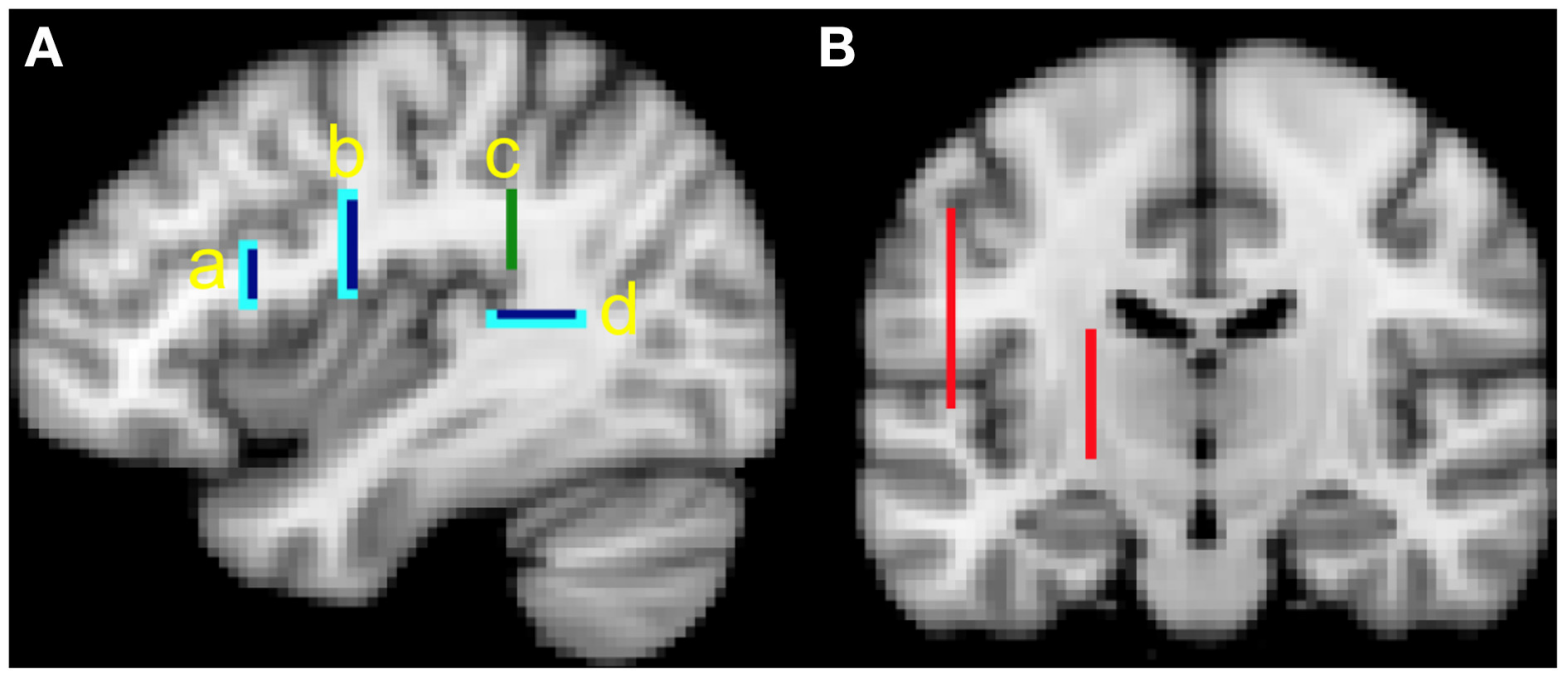
**(A)** Seed (green bar), target (dark blue bars) and termination (light blue bars) masks, *x* = −38. The primary analysis involving tractography between white matter in the posterior superior temporal gyurs (STG) and premotor cortex implicate masks b, c and d. The secondary analysis involving tractography between white matter in the posterior STG and pars opercularis implicate masks a, c, and d; **(B)** exclusion masks (red bars), *y* = −16.

The ROIs (see Figure 1) include:

1. One seed mask from which tractography proceeds:
  This mask is located in the white matter of the AF, where the Sylvian Fissure begins to curve superiorly. This allows us to track AF fibers that proceed anteriorly toward the frontal regions, as well as fibers that proceed inferiorly toward the temporal lobe. The ROI was drawn in a single coronal slice at *y* = −38, extending from *x* = −30 to −42 (left hemisphere) or *x* = 30 to 42 (right hemisphere), and *z* = 20 to 34 (Figure 1A, green bar).
2. Two target masks from which only pathways that reach it are kept:
  - The first target mask is located in white matter just posterior to Heschl's gyrus in the posterior aspect of the STG. This was drawn in a single horizontal slice at *z* = 10, extending from *x* = −32 to −44 (left hemisphere) or *x* = 32 to 44 (right hemisphere), and *y* = −36 to −50 (Figure 1A, dark blue bars), and appears roughly equivalent to the posterior STG ROI used in the study by Loui et al. (2009).
  - The second target mask is located in the white matter underlying the precentral gyrus. This was drawn in a single coronal slice at *y* = −6, extending from *x* = −26 to −42 (left hemisphere) or *x* = 26 to 42 (right hemisphere), and *z* = 16 to 32. (Figure 1A, dark blue bars) and is posterior to the pars opercularis ROI used in the study of Loui et al. (2009).
3. Two termination masks from which pathways going beyond are eliminated. These ROIs include voxels that are just anterior and inferior to the target ROIs (Figure 1A, light blue bars).
4. Two exclusion masks from which entire pathways are removed should they enter these voxels. These ROIs remove pathways that may branch off the AF, and are not part of it. They are located medial and lateral to the masks described above. The first ROI was drawn on a sagittal slice at *x* = −50 (left hemisphere) or *x* = 50 (right hemisphere), extending from *y* = −10 to −62 and *z* = 6 to 44. The second ROI was drawn on a sagittal slice at *x* = −22 (left hemisphere) or *x* = 22 (right hemisphere), extending from *y* = 4 to −20, and *z* = −4 to 20 (Figure 1B, red bars).

The three tracking methods yielded connectivity maps for each AF and each participant. Each voxel represents the number of streamlines passing through it. To obtain tract volume, each non-zero voxel was multiplied by the voxel size. For each tracking result we calculated the cumulative histogram multiplied with the voxel volume, which represents the tract volume at a particular threshold. We evaluated the data at two specific thresholds, 10 and 100, where 10 for example, represents the number of streamlines that pass through a voxel. This allows us to illustrate what group differences would have been found at a set threshold with each of the three tracking methods. Importantly, the aim of our approach is to show that the main findings of interest occur independent of thresholding.

For each of the three tractography analyses, we performed a repeated measures ANOVA with group (amusic; control) and hemisphere (left; right) as factors.

### Secondary MRI analysis

#### Tracking to pars opercularis

To better compare our findings with those of Loui et al. (2009), we also performed tractography to a white matter mask underlying pars opercularis. This mask was drawn in a single coronal slice at *y* = 14, extending from *x* = −30 to 46 (left hemisphere) or *x* = 30 to 46 (right hemisphere), and *z* = 14 to 22 (Figure 1A). A termination mask just anterior to this pars opercularis mask was used. We used the Harvard-Oxford Atlas in FSL as a guide to localize the pars opercularis. We kept the target mask in posterior STG the same since it is similar to that used by Loui et al. (2009). All other parameters were unchanged. Thus, the only difference in this analysis is the use of a mask that underlies the white matter of pars opercularis, instead of a mask that underlies the white matter of the premotor cortex. We performed tractography using the probabilistic 2-fiber and deterministic models and compared results thresholded at 10 and 100.

#### Tracking the posterior segment of the AF

Loui et al. (2009) also suggest the right posterior STG is “lacking in its connectivity specificity toward the frontal lobe.” We tested this hypothesis by performing tractography of the posterior segment of the AF. Using identical masks from the original analysis, we instead seeded from the white matter mask underlying the posterior STG (originally one of the target masks), to the white matter AF mask as a target (originally the seed mask). We additionally created a termination mask just anterior to the white matter AF mask. All other parameters were unchanged. We performed tractography using the probabilistic 2-fiber and deterministic models and compared results thresholded at 10 and 100.

## Results

Results of the analysis on the full data set and a subgroup (i.e., where amusic and control individuals were matched on all variables, see Materials and Methods) show the same finding (see Supplementary Results). Therefore, analysis with the full data set is presented.

**Figure 2:**
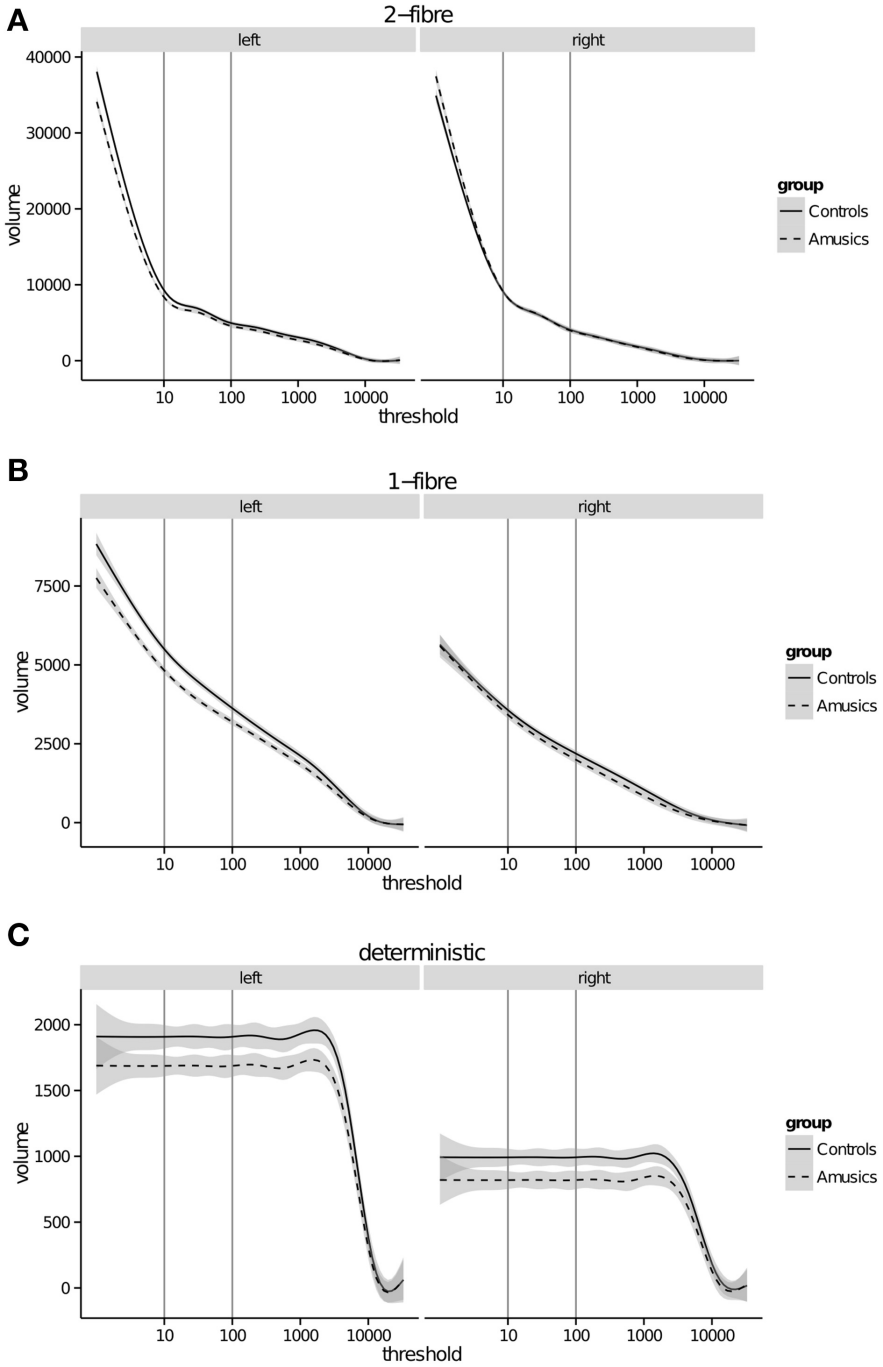
**Arcuate fasciculus tract volume (mm^3^) (y-axis) calculated at every threshold (x-axis) for **(A)** probabilistic 2-fiber model; **(B)** probabilistic 1-fiber model; **(C)** deterministic model**. Gray bars represent 95% confidence intervals.

The tract volume was calculated at every threshold (Figure 2) to visualize how these two variables relate to one another. Since the deterministic algorithm explores only one path (i.e., the best estimated streamline), tract volume is identical across thresholds within each subject, up to a maximum where it then falls to zero. This drop occurs at different and discrete thresholds across subjects, whereas the average suggests a smooth drop off (Figure 2C).

**Figure 3:**
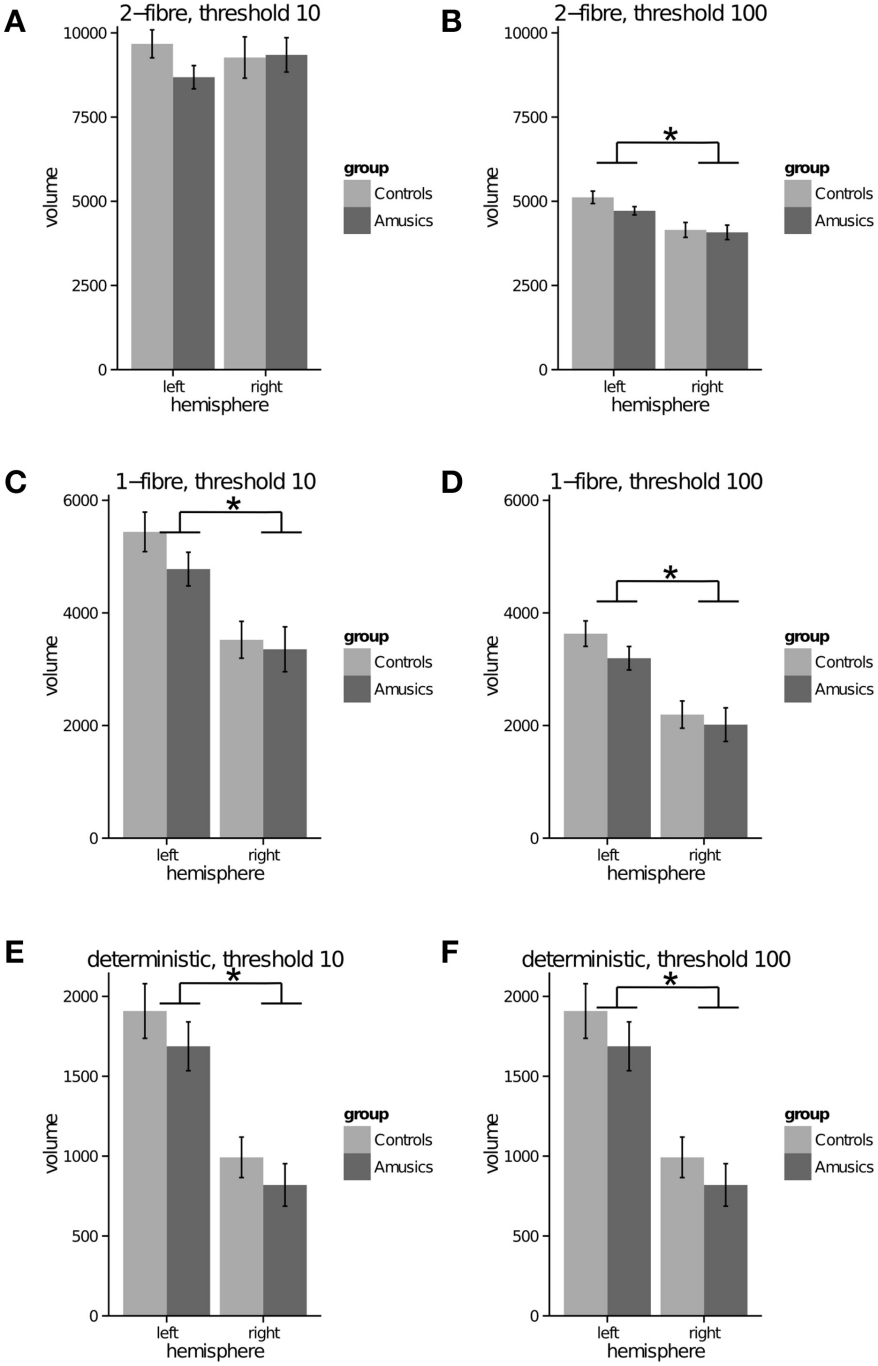
**Arcuate fasciculus (to premotor cortex) tract volume (mm^3^) (y-axis) in left and right hemispheres (x-axis) for control and amusic individuals**. **(A)** probabilistic 2-fiber model, data thresholded at 10; **(B)** probabilistic 2-fiber model, data thresholded at 100; **(C)** probabilistic 1-fiber model, data thresholded at 10; **(D)** probabilistic 1-fiber model, data thresholded at 100; **(E)** deterministic model, data thresholded at 10; **(F)** deterministic model, data thresholded at 100. * represents a significant main effect of hemisphere, *p* < 0.001.

**Figure 4:**
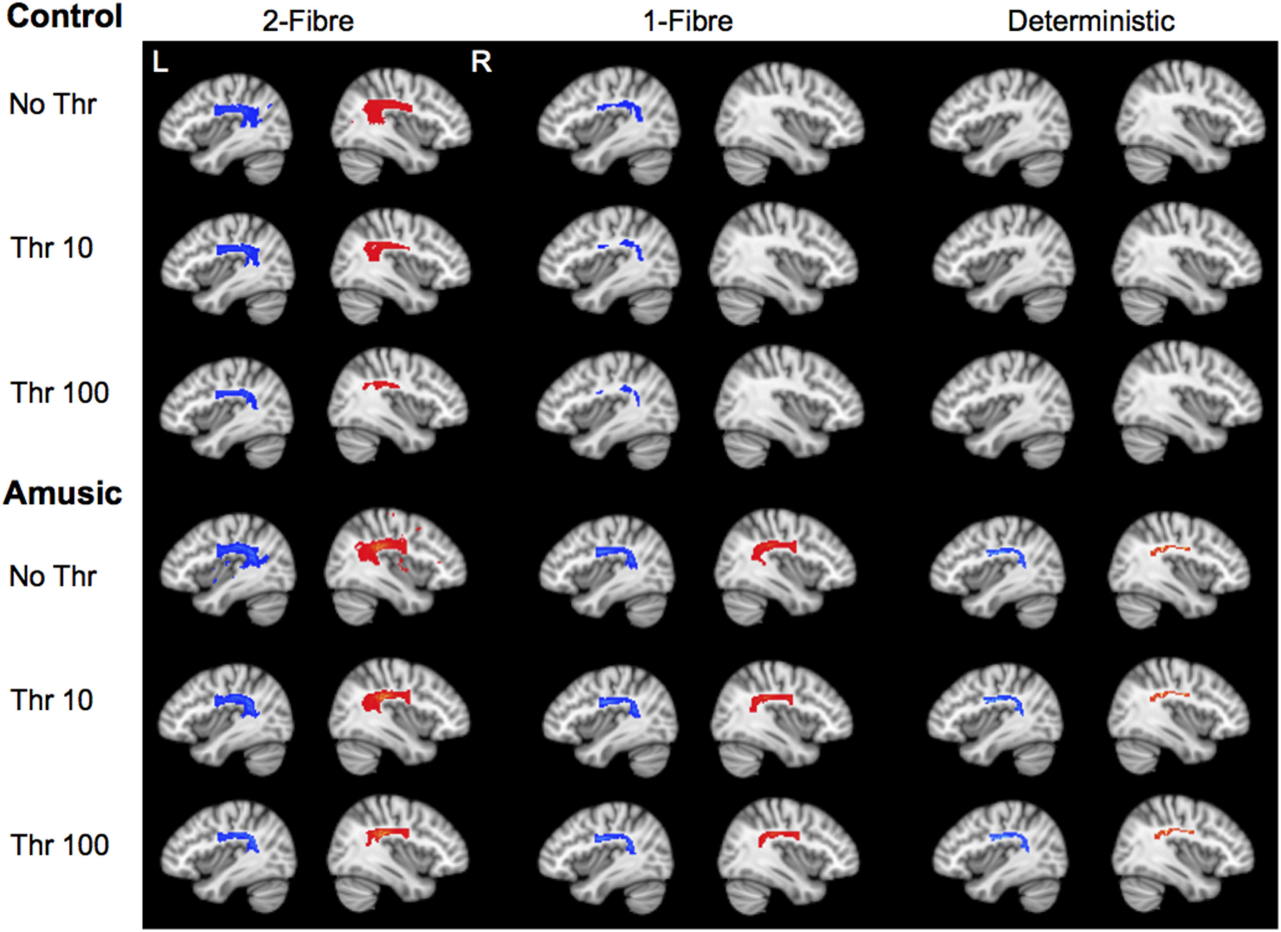
**Tractography results for one control and one amusic individual, for each tractography algorithm**. Results are presented at no thresholding, threshold of 10, and threshold of 100, overlaid on the standard MNI template (thresholding scale from 0 to 24500). L, left, *x* = −38; R, right, *x* = 38. In this sample control individual, fiber tracking failed in the right hemisphere for the 1-fiber model, and in both hemispheres in the deterministic model.

Probabilistic model—2 fiber: For both thresholds (10, 100), the repeated measures ANOVA showed no significant effect of group (*p* = 0.386; *p* = 0.311), a significant effect of hemisphere with greater tract volume in the left than right AF, for only the threshold at 100 [*p* = 0.773; *F*_(1, 49)_ = 32.82 *p* < 0.001], and no significant interaction (*p* = 0.234; *p* = 0.251) (see Figures 3A,B). In the unthresholded data, the AF could be tracked in all participants in both groups and hemispheres. (See Figure 4 for representative data from one control and one amusic individual).

Probabilistic model—1 fiber: For both thresholds (10, 100), the repeated measures ANOVA showed no significant effect of group (*p* = 0.303; *p* = 0.293), a significant effect of hemisphere with greater tract volume in the left than right AF, [*F*_(1, 49)_ = 34.87, *p* < 0.001; *F*_(1, 49)_ = 46.19, *p* < 0.001], and no significant interaction (*p* = 0.388; *p* = 0.506) (see Figures 3C,D). In the unthresholded data, the AF could not be tracked in the following: left hemisphere for one amusic participant; right hemisphere for two control participants. (See Figure 4 for representative data from one control and one amusic individual).

Deterministic: For both thresholds (10, 100), the repeated measures ANOVA showed no significant effect of group (*p* = 0.257; *p* = 0.257), a significant effect of hemisphere with greater tract volume in the left than right AF [*F*_(1, 49)_ = 57.59, *p* < 0.001; *F*_(1, 49)_ = 57.59, *p* < 0.001], and no significant interaction (*p* = 0.839; *p* = 0.839) (see Figures 3E,F). Note that data for both thresholds are identical since the deterministic algorithm does not explore different paths, but the same one regardless of threshold. In the unthresholded data, the AF could not be tracked in the following: left hemisphere for two amusic participants; left hemisphere for two control participants; right hemisphere for five amusic participants; right hemisphere for three control participants. (See Figure 4 for representative data from one control and one amusic individual).

### Secondary MRI analysis

#### Tracking to pars opercularis

**Figure 5:**
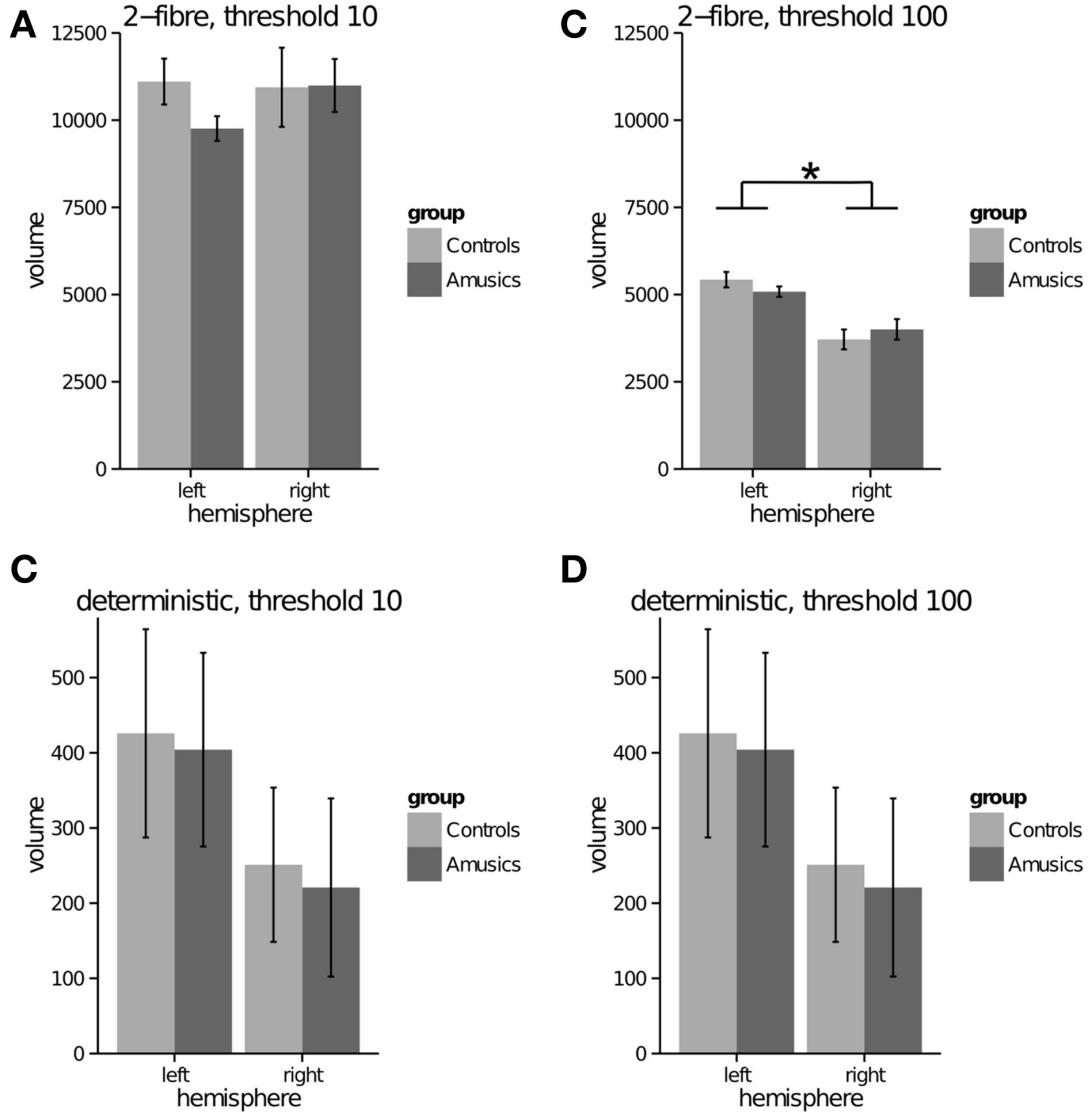
**Arcuate fasciculus (to pars opercularis) tract volume (mm^3^) (y-axis) in the left and right hemispheres (x-axis) for control and amusic individuals**. **(A)** probabilistic 2-fiber model, data thresholded at 10; **(B)** probabilistic 2-fiber model, data thresholded at 100; **(C)** deterministic model, data thresholded at 10; **(D)** deterministic model, data thresholded at 100. * represents a significant main effect of hemisphere, *p* < 0.001.

Probablistic model—2 fiber: For both thresholds (10, 100), the repeated measures ANOVA showed no significant effect of group (*p* = 0.445; *p* = 0.918), a significant effect of hemisphere with greater tract volume in the left than right AF, for only the threshold at 100 [*p* = 0.549; *F*_(1, 49)_ = 44,935, *p* < 0.001], and no significant interaction (*p* = 0.338; *p* = 0.136) (see Figures 5A,B). In the unthresholded data, the AF could be tracked in all participants in both groups and hemispheres.

Deterministic: For both thresholds (10, 100), the repeated measures ANOVA showed no significant effect of group (*p* = 0.835; *p* = 0.835), no significant effect of hemisphere (*p* = 0.147; 0.147), and no significant interaction (*p* = 0.972; *p* = 0.972) (see Figures 5C,D). In the unthresholded data, the AF could not be tracked in more than half the number of control and amusic participants: left hemisphere for 16 amusic participants; left hemisphere for 16 control participants; right hemisphere for 19 amusic participants, right hemisphere for 20 control participants.

#### Tracking the posterior segment of the AF

Probablistic model—2 fiber: For both thresholds (10, 100), the repeated measures ANOVA showed no significant effect of group (*p* = 0.904; *p* = 0.463), a significant effect of hemisphere with greater tract volume in the left than right posterior AF segment [*F*_(1, 49)_ = 6.67, *p* < 0.05; *F*_(1, 49)_ = 12.12, *p* < 0.001], and no significant interaction (*p* = 0.733; *p* = 0.495). In the unthresholded data, the posterior segment of the AF could be tracked in all participants in both groups and hemispheres.

Deterministic model: For both thresholds (10, 100), the repeated measures ANOVA showed no significant effect of group (*p* = 0.186; *p* = 0.186), a significant effect of hemisphere with greater tract volume in the left than right posterior AF segment [*F*_(1, 49)_ = 7.90, *p* = 0<0.05; *F*_(1, 49)_ = 7.90, *p* = 0<0.05], and no significant interaction (*p* = 0.192; *p* = 0.192). In the unthresholded data, the AF could not be tracked in: right hemisphere for 2 amusic participants, right hemisphere for 1 control participant.

## Discussion

Findings from this study demonstrate that for the group of individuals tested, the volume of the AF does not significantly differ between amusics and controls, for either left or right hemispheres. This finding was the same across all three tracking algorithms and at low and high thresholds. Importantly however, we found that the type of tracking algorithm does influence whether the AF can be detected. Using probabilistic tractography that models two fiber populations, the AF could be tracked in all individuals, in both hemispheres. In contrast, the deterministic model failed to detect the left and right AF in some amusic and control individuals. Findings from this study provide an alternate and methodologically based explanation for why the AF may not have been detected in prior studies.

In particular, one study showed that the right AF connecting posterior STG with pars opercularis was unidentifiable in nine of the 10 amusic individuals (Loui et al., 2009). We did not replicate these findings in our larger group of 25 amusics and 26 controls. There are several reasons that may explain this discrepancy. First, the acquisition parameters were different. For example, Loui et al. (2009) acquired 30 diffusion directions at a voxel resolution of 2.5 mm^3^. In the present study, we acquired 60 directions at a voxel resolution of 2.3 mm^3^. In general, the greater the number of diffusion directions, the better the estimate of the diffusion tensor, and hence the ability to resolve mulitiple fibers (Behrens et al., 2007). The angular resolution will increase, thus allowing one to differentiate tracts that cross at a shallower angle. Since the AF crosses with the corticospinal tract (CST), the greater number of directions allows for a better estimate of the tensors in these crossing-fiber voxels. The modeling of multiple fibers then allow the investigator to better resolve and thus detect the AF from other competing fiber systems such as the CST. Model-free approaches such as HARDI (high angular resolution diffusion imaging) measure diffusion at many orientations and thus also allow one to better resolve multiple fibers within a voxel. This is achieved through the use of a high number of diffusion directions (60–100) with *b*-values higher than the 1000 s/mm2 implemented in the present study (Campbell and Pike, 2014).

Second, we used different data analysis software (MedINRIA vs. FSL). To ensure comparability of findings between deterministic and probabilistic tractography within the present study, we used FSL; MedINRIA is only capable of performing deterministic tractography. Related, it is not obvious how standardized ROIs can be implemented in MedINRIA (version 1.7). In this case, the ROIs for each subject may slightly differ in anatomical space.

Third, our approach also differed in the masks used to virtually dissect the AF. We used white matter masks and delineated the bulk of the AF. This was to avoid the controversies of defining gray matter regions that connects the AF (as discussed in the Introduction). As such, we implemented a seed in the white matter of the AF and tracked anteriorly and posteriorly, as per prior methods (Giorgio et al., 2010). In contrast, another commonly used approach is to define two seeds, regions A and B, track between A to B then B to A, and combine these results. This takes into consideration that connectivity drops with distance from the seed mask. However, it is unclear how streamlines common to both A to B, and B to A, are counted once in the overall analysis. Our approach selects a region in between regions A and B and from here, simultaneously track to A and B.

Furthermore, we also used termination and exclusion masks to restrict tractography. This was done to meet our criteria of dissecting the bulk of the AF. Tractography of the AF that does not impose restrictions may yield streamlines that extend beyond the bulk of the tract, such as to the parietal and contralateral cortex (Loui et al., 2009). It can be argued that some of these streamlines could be peripheral branches of the AF and should be included in further data analyses. This was not the goal of our study.

We also chose to dissect the bulk of the AF using a target in posterior STG, in contrast to the posterior MTG as some prior work have done (Glasser and Rilling, 2008; Loui et al., 2009). This decision is based on evidence from monkey tracer studies that show the AF as monosynaptically connecting posterior STG with inferior frontal regions (reviewed in Petrides, 2013). It is argued that one cannot be certain other regions found linked by the AF, as suggested by human tractography studies, are not artifacts of the methods (Frey et al., 2008; Petrides, 2013). There are several fiber systems coursing through the same region as the AF that are difficult to segregate with current tractography approaches (Campbell and Pike, 2014). Fiber tracts steming from the posterior MTG may encompass the middle longitudinal fasciculus and AF (Petrides, 2013). Thus, it is possible that the portion of the AF thought to stem from the posterior MTG is in fact composed of the middle longitudinal fasciculus and AF. Related, Loui et al. (2009) were able to bilaterally track from posterior MTG to pars opercularis in all amusic individuals. It is also possible that this tract includes the middle longitudinal fasciculus with other branches of the superior longitudinal fasciculus such as II and III, which course through the same regions and may be unaffected in amusia.

The fourth major difference between studies is that there may have been differences in the samples of amusic individuals even though participants in both studies were classified according to the MBEA (Peretz et al., 2003). There is evidence the amusia phenotype is not homogenous. A small sample of individuals also show impairments related to working memory and mental rotation (Williamson and Stewart, 2010; Williamson et al., 2011). There is also heterogeneity related to performance on pitch production vs. pitch perception tasks (including pitch direction detection) (Williamson et al., 2012). Thus, it is possible for Loui et al. (2009) to detect changes in the AF if: (1) their sample subjects were more homogeneous in having deficits related to pitch production/perception, (2) these deficits were consistent (i.e., minimal inter-individual variability), and (3) we assume these deficits relate to the structural integrity of the AF. If the subjects in the present study were more heterogeneous (i.e., have deficits in other domains such as working memory, and have high inter-individual variability in performance even within a domain of testing), it is possible that the structural integrity of the AF would be relatively more intact since the anatomical abnormalities related to our subject characteristics may be distributed. Thus, we would not be sensitive in detecting AF volume differences between amusics and controls.

Despite these differences, however, we have evidence that there is diminished sensitivity in detecting the AF when deterministic tractography that does not model crossing fibers is employed. In our primary analysis that tracked the AF to the white matter underlying premotor cortex, the AF could not be tracked in the left hemisphere for two amusic and two control participants, and in the right hemisphere for five amusic and three control participants. Even more striking are findings from our secondary analysis that tracked to the white matter underlying pars opercularis. Here, the AF could not be tracked in the left hemisphere for 16 amusic and 16 control participants, and in the right hemisphere for 19 amusic and 20 control participants. Together, these deterministic findings are supported by prior studies which also used a similar algorithm and report a failure to detect the left or right AF in some healthy control subjects (Catani et al., 2007; Glasser and Rilling, 2008; Lebel and Beaulieu, 2009; Kaplan et al., 2010; Thiebaut de Schotten et al., 2011). Thus, despite different methodologies, there is consistency in the finding of reduced sensitivity for detecting the AF when using deterministic methods without modeling crossing fibers. For example, it may be difficult to track the AF through regions where the corticospinal tract crosses and thus deterministic tractography can fail and a false negative is reported. In particular, there may be voxels of even higher uncertainty in the inferior frontal region that makes it difficult to track using deterministic approaches that do not model crossing fibers. In contrast, probabilistic tractography that models 2 fiber populations successfully tracked the AF in all participants, to both the white matter underlying premotor cortex and the pars opercularis. Findings from both analyses were very similar in the magnitude of tract volume derived (compare Figures 4, 5).

On the other hand, probabilistic tractography may be prone to false positives. In the field, there is at present no criterion to select an appropriate threshold. Thresholding allows us to rule out spurious connections, defined by voxels where there is a higher degree of uncertainty with regard to the presence of a fiber orientation. This is particularly relevant for probabilistic tractography since there are an infinite number of possible paths through the data. In contrast by definition, there is only one path that is delineated in deterministic tractography. This difference is illustrated in our data set in two ways. First, there is a difference in magnitude of the volume measure across the three tracking algorithms (see Figure 2, y-axis values). Second, tract volume at low and high thresholds for deterministic tracking is identical, i.e., additional deterministic streamlines do not explore the tract beyond the initial streamlines because they follow the same path. This is clearly seen in Figure 2C where volume is relatively constant at all thresholds once the “deterministic” tract has been established. In contrast, the 1-fiber model shows an exponential relationship between tract volume and threshold when excluding thresholds lower than five. The 2-fiber model shows a marked deviation from the exponential relationship at a threshold around 20. Here tract volume increases dramatically as soon as lower-connectivity voxels are included.

In probabilistic tractography, one might assume that voxels of higher uncertainty are included at a lower connectivity threshold, because they are less often traversed by streamlines. This is demonstrated in Figure 4 where the unthresholded images in both control and amusic individuals (2-fiber model) show spurious (e.g., false positive) voxels in non-AF regions with a non-zero connectivity. This is in spite of strict exclusion masks designed to restrict tracking to the AF. Furthermore, the closer these low-connectivity voxels are to the bulk AF the more difficult it is to determine what proportion of those voxels are false positives, and what proportion might be considered to be actual branches of the AF. These branches may be small and variable, and thus are detected but with a higher degree of uncertainty. Global histogram-based thresholding cannot dissociate between these options, because it does not take the proximity and connection to the core of the tract into account. Therefore, the 2-fiber model may facilitate tracking by proceeding through regions of noise and crossing fibers, but the interpretation of this data at lower thresholds may be problematic (e.g., false positives) (Campbell and Pike, 2014). The histograms in this work indicate that the super-exponential increase of tract volumes with thresholds lower than 10–20 might form a lower bound on a reasonable threshold (Figure 2A). Looking at the entire distribution across different thresholds may be one approach to gain a better understanding of the data.

This study also found the expected left greater than right AF volume that has been attributed to language lateralization (Nucifora et al., 2005; Parker et al., 2005; Powell et al., 2006; Barrick et al., 2007; Catani et al., 2007; Glasser and Rilling, 2008). This finding was true for the probabilistic 1-fiber and deterministic models. There was no main effect of hemisphere for the probabilistic 2-fiber model at the lower threshold of 10. A significant left-right difference was detected at a higher threshold of 100, which suggests that the laterality is more prominent in the core of the AF. That is, the laterality effect does not occur at the level of the AF branches, delineated by voxels of higher uncertainty (i.e., low connectivity). An alternative is that due to the mixing of noise and signal in these lower connectivity voxels, potential left-right differences are obscured.

Returning to the biological basis of congenital amusia, our findings suggest that the AF is not affected in the individuals tested for this study. We also performed a secondary analysis to determine whether differences could be found in the posterior segment of the AF, as per prior work (Loui et al., 2009). We did not find any evidence that tract volume in the posterior segment differed between groups in our study. The impairments manifested in amusia are quite subtle, as these individuals have no other neurological impairment. Thus, one might also expect nuanced differences in brain anatomy, rather than the absence of a complete major fiber pathway. Evidence from prior work converges on the finding of anatomical and functional abnormalities in the inferior frontal cortex and posterior STG (Hyde et al., 2006, 2007, 2011; Albouy et al., 2013) (see Figure 12 in Albouy et al., 2013). Our findings suggest that these abnormalities may be unrelated to a major deficit in an anatomical pathway that might connect these areas. Alternatively, the current resolution of diffusion MRI may be unable to detect possible changes in the AF or other fiber tracts that may be affected.

## Conclusions

In summary, the present study demonstrates the choice of tractography algorithm determines the extent to which the AF can be detected in the brains of healthy controls and amusic individuals. All of the amusics tested in this study have an AF in the right hemisphere. This research suggests that when tracking the AF, false negatives may be reduced when probabilistic tractography is implemented and crossing fibers modeled, though this may occur at the expense of false positives if no adequate threshold is chosen.

### Conflict of interest statement

The authors declare that the research was conducted in the absence of any commercial or financial relationships that could be construed as a potential conflict of interest.
