# Supplementary material for: Detection of the arcuate fasciculus in congenital amusia depends on the tractography algorithm
Source: Front Psychol. 2015 Jan 21;6:9. doi: 10.3389/fpsyg.2015.00009 (PMC4300860; doi:10.3389/fpsyg.2015.00009)
Supplement: Supplementary file 1 [file DataSheet1.DOCX]

**Supplemental Methods**

Supplementary Table 1: Demographics

|  | Group | N | Age | NART | Digit Span | Years musical training | Years formal education | MBEA Scale | MBEA Contour | MBEA Interval | Pitch composite |
| --- | --- | --- | --- | --- | --- | --- | --- | --- | --- | --- | --- |
| μ | Amusic | 14 | 50.14 | 41.86 | 21.07 | 4.71 | 15.64 | 18.71 | 19.57 | 18.21 | 56.50 |
| σ |  |  | 10.42 | 4.72 | 3.32 | 0.47 | 1.15 | 2.64 | 2.93 | 2.64 | 6.86 |
| μ | Control | 15 | 49.07 | 44.4 | 20.60 | 4.47 | 14.67 | 27.20 | 27.93 | 27.60 | 82.73 |
| σ |  |  | 12.04 | 2.20 | 3.62 | 0.51 | 2.22 | 2.24 | 2.15 | 2.23 | 5.57 |
|  |  |  |  |  |  |  |  |  |  |  |  |
| T |  |  | 0.26 | 1.88 | 0.36 | 1.35 | 1.47 | 9.34 | 8.80 | 10.38 | 11.34 |
| p |  |  | 0.80 | 0.07 | 0.72 | 0.19 | 0.15 | <0.001 | <0.001 | <0.001 | <0.001 |

N, number of participants; NART, National Adult Reading Test; MBEA, Montreal Battery of Evaluation of Amusia.

**Supplemental Results**

These results are based on a sample of amusic (n=14) and control (n=15) individuals who were matched on all variables.

The voxel volume was calculated at every threshold (Fig 1) to visualize how these two variables relate to one another.


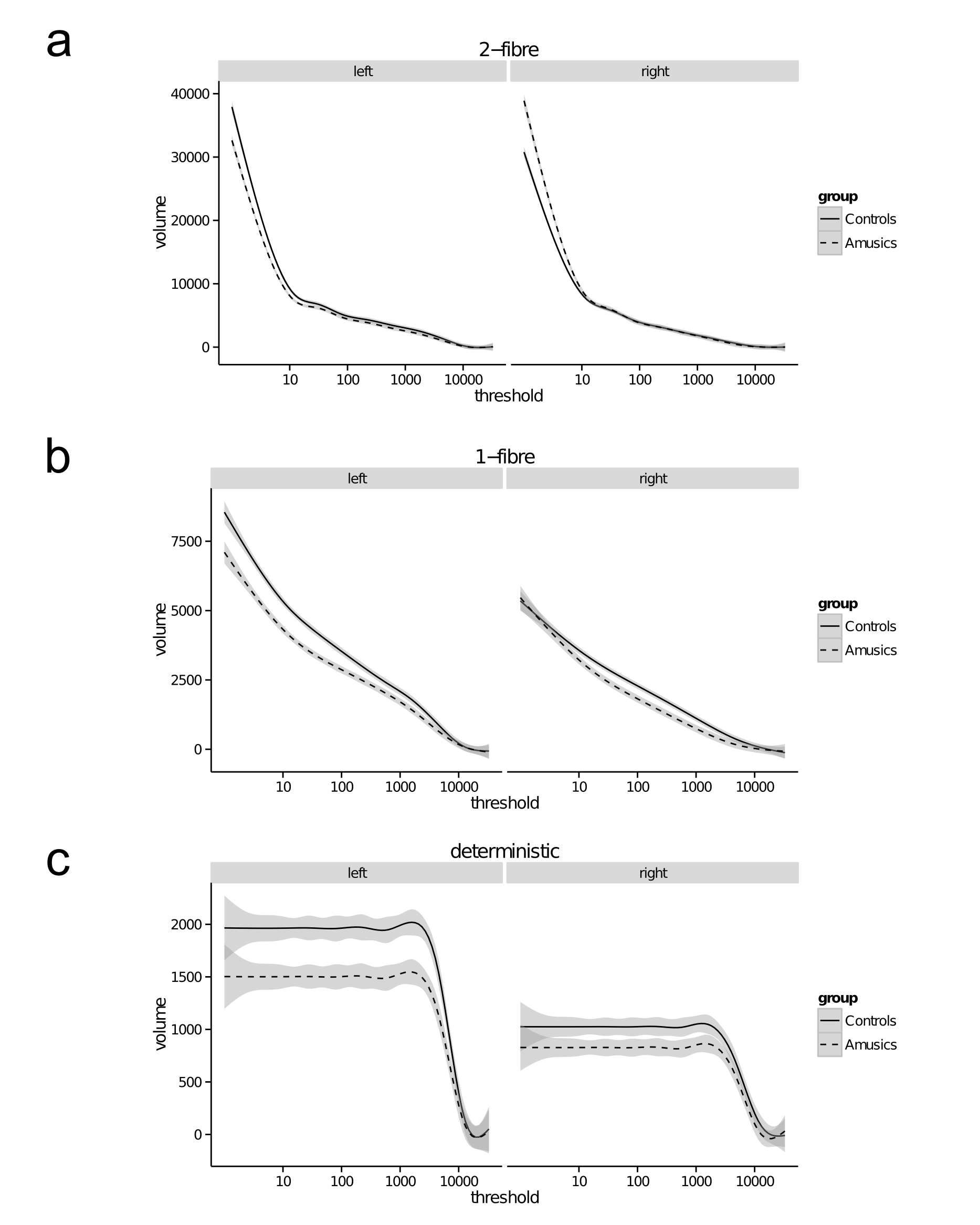


**Figure 1:** Arcuate fasciculus tract volume (mm^3^) (y-axis) calculated at every threshold (x-axis) for a) probabilistic 2-fiber model; b) probabilistic 1-fiber model; c) deterministic model.

Probabilistic model – 2 fiber: For each threshold (10, 100), the repeated measures ANOVA showed no significant effect of group (p=0.663; p=0.365), significant effect of hemisphere for only the threshold at 100 (p=0.823; F(1,27=16.82), p<0.001), and no significant interaction (p=0.122; p=0.294) (Fig 2a,b). In the unthresholded data, the AF was tracked in all participants in both groups and hemispheres.

Probabilistic model – 1 fiber: For each threshold (10, 100), the repeated measures ANOVA showed a significant main effect of hemisphere (F (1,27)=12.70, p<0.001; F (1,27)=17.91, p<0.001) with greater tract volume in the left than right. There was no significant main effect of group (p=0.130; p=0.097) or interaction (p=0.450; p=0.694) (see Fig 2c,d). In the unthresholded data, the AF could not be tracked in the following: left hemisphere for one amusic participant; right hemisphere for one control participant.

Deterministic: For each threshold (10, 100), the repeated measures ANOVA showed a significant main effect of hemisphere (F(1,27)=22.38, p<0.001; F(1,27)=22.38, p<0.001) with greater tract volume in the left than right. There was no significant main effect of group (p=0.135; p=0.135) or interaction (p=0.445; p=0.445) (see Fig 2e,f). In the unthresholded data, the AF could not be tracked in the following: left hemisphere for two amusic participants; left hemisphere for one control participant; right hemisphere for two amusic participants; right hemisphere for one control participant.


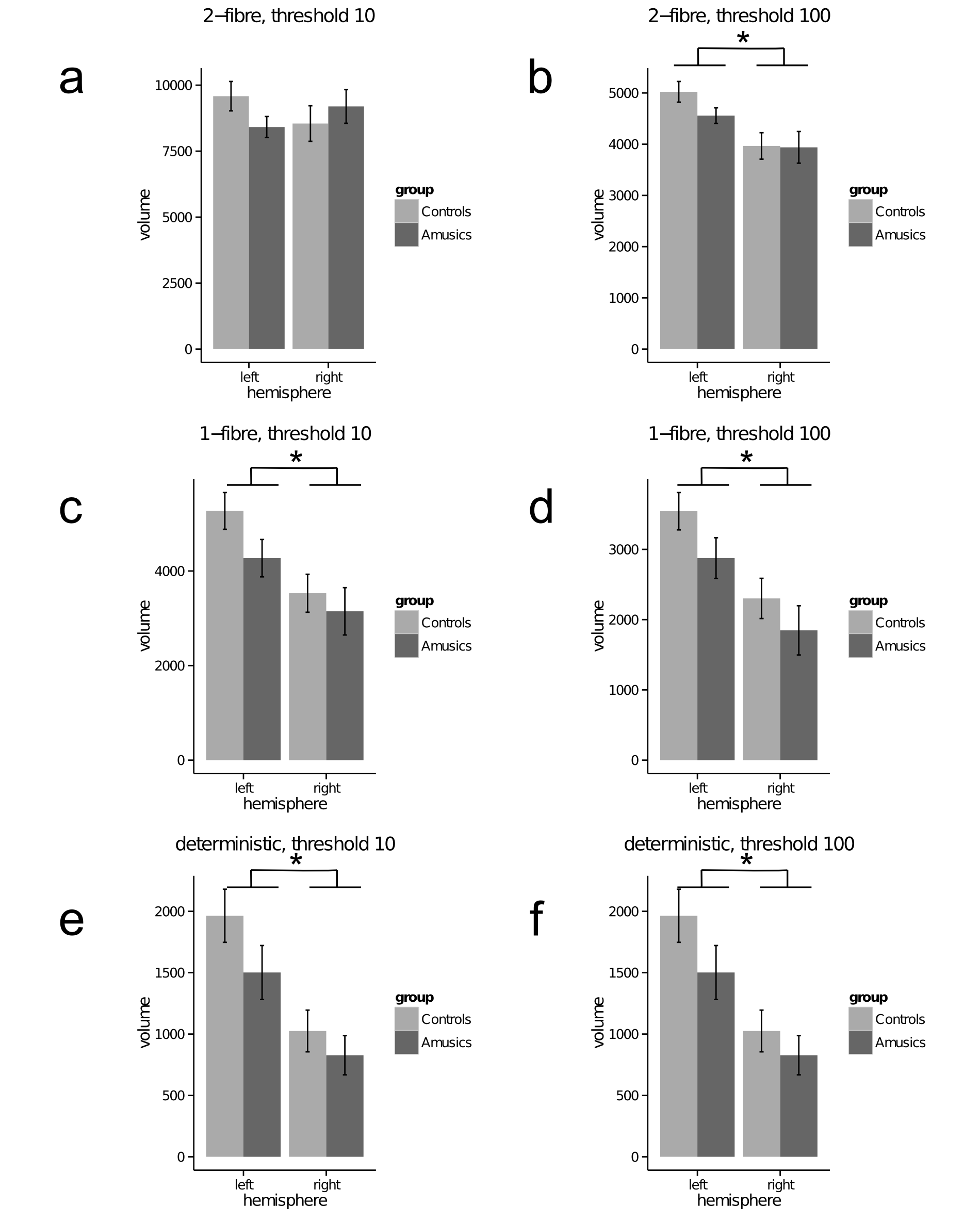


**Figure 2:** Arcuate fasciculus tract volume (mm^3^) (y-axis) in left and right hemispheres (x-axis) for control and amusic individuals: a) probabilistic 2-fiber model, data thresholded at 10; b) probabilistic 2-fiber model, data thresholded at 100; c) probabilistic 1-fiber model, data thresholded at 10; d) probabilistic 1-fiber model, data thresholded at 100; e) deterministic model, data thresholded at 10; f) deterministic model, data thresholded at 100.
